# Supplementary material for: Exploring the Long-Term Tissue Accumulation and Excretion of 3 nm Cerium Oxide Nanoparticles after Single Dose Administration
Source: Antioxidants (Basel). 2023 Mar 21;12(3):765. doi: 10.3390/antiox12030765 (PMC10045098; doi:10.3390/antiox12030765)
Supplement: Supplementary file 1 [file antioxidants-12-00765-s001.zip › antioxidants-2280658-supplementary.pdf]

Exploring the Long Term Tissue Accumulation and Excretion of 3 nm Cerium Oxide Nanoparticles after Single Dose Administration

Lena M. Ernst[1], Laura Mondragon [1, 2], Joana Ramis[1], Muriel F. Gustà[3,4], Tetyana Yudina[3], Eudald Casals [1,5], Neus G. Bastús [3,4], Guillermo Fernández-Var [6], Gregori Casals[6,7], Wladimiro Jiménez [6,8], Victor Puntes [1,3,4,9]*

[1] Vall d'Hebron Research Instiute (VHIR), 08035 Barcelona, Spain
[2] Josep Carreras Leukaemia Research Institute (IJC), Badalona, Spain
[3] Instiut Català de Nanociència I Nanotecnologia (ICN2), CSIC, The Barcelona Institute of Science and Technology (BIST), Campus UAB, Bellaterra,Barcelona, Spain
[4] Networking Research Centre for Bioengineering, Biomaterials, and Nanomedicine (CIBER-BBN), Instituto de Salud Carlos III, Madrid, Spain.
[5] School of Biotechnology and Health Sciences, Wuyi University, Jiangmen, 529020, China
[6] Service of Biochemistry and Molecular Genetics, Hospital Clinic, Centro de Investigación Biomédica en Red de Enfermedades Hepáticas y Digestivas (CIBERehd), Institut d'Investigacions Biomèdiques August Pi i Sunyer (IDIBAPS), Barcelona, Spain
[7] Department of Fundamental Care and Medical-Surgical Nursing, Universitat de Barcelona, Spain.
[8] Departament de Biomedicina, Universitat de Barcelona, Spain.
[9] Institució Catalana de Recerca i Estudis Avançats (ICREA),Barcelona, Spain

** Corresponding autor: victor.puntes@vhir.org*

Content:

Table S1. Cerium content by organ and days, given in µg Ce/g tissue and % of injected dose.

Figure S1. Cerium excretion in µg Ce/g tissue between day 1 and day 100.

Figure S2. Mice weight evolution up to 30 days after nanoceria administration.

Figure S3. Dissolution of CeO_2 Nanoparticles at pH 1.7.

Figure S4. PourBoix diagram.

Figure S5. The liver and spleen are major targets for nanoparticles in CCl4-treated rats.

Extended Materials and Methods.

Table S1. Cerium content by organ and days, given in µg Ce/g tissue and % of injected dose.

Organ	*Time post-injection*	*Ce quantity per organ (µg/g tissue)*		*Ce quantity per organ (%ID)*	
		Mean	Standard deviation	Mean	Standard deviation
Liver	24 h	89.818	15.763	82.46	7.45
	9 days	50.827	8.351	50.67	7.78
	30 days	47.727	6.637	46.03	2.53
	100 days	40.945	5.535	40.00	2.44
Spleen	24 h	48.969	10.595	5.05	0.57
	9 days	47.688	10.035	4.51	0.54
	30 days	50.448	17.160	4.75	0.70
	100 days	38.814	2.834	4.08	0.62
Kidney	24 h	5.064	1.927	0.629	0.178
	9 days	2.868	1.358	0.385	0.138
	30 days	3.880	0.792	0.508	0.091
	100 days	3.603	1.041	0.452	0.106
Lung	24 h	1.006	1.016	0.189	0.076
	9 days	0.962	0.766	0.140	0.119
	30 days	0.939	0.318	0.120	0.040
	100 days	0.638	0.239	0.097	0.027
Brain	24 h	0.033	0.020	0.009	0.005
	9 days	0.031	0.006	0.016	0.015
	30 days	0.017	0.005	0.005	0.002
	100 days	0.025	0.007	0.008	0.002
Lymph Nodes	24 h	2.333	1.357	0.098	0.122
	9 days	0.507	0.320	0.018	0.020
	30 days	4.111	1.912	0.368	0.347
	100 days	2.149	0.821	0.029	0.016
Ovary	24 h	3.386	0.916	0.524	0.082
	9 days	2.466	1.208	0.359	0.112
	30 days	2.730	1.523	0.390	0.176
	100 days	2.135	2.037	0.377	0.358
Bone Marrow	24 h	1.750	0.879	0.036	0.022
	9 days	1.323	1.668	0.035	0.047
	30 days	1.202	0.215	0.045	0.017
	100 days	1.065	0.902	0.016	0.007
Faeces	24 h	2.319	0.193	0.607	0.193
	9 days	0.100	0.061	0.100	0.061
	30 days	0.060	0.027	0.060	0.027
	100 days	0.057	0.016	0.057	0.016
Urine	24 h	0.075	0.044	0.002	0.001
	9 days	0.047	0.025	0.002	0.001
	30 days	0.035	0.001	0.001	0.000
	100 days	0.006	0.006	0.001	0.000

Figure S1. Mice weight evolution up to 30 days after nanoceria administration. n = 20, 15 and 10 at days 1, 9 and 30, respectively.

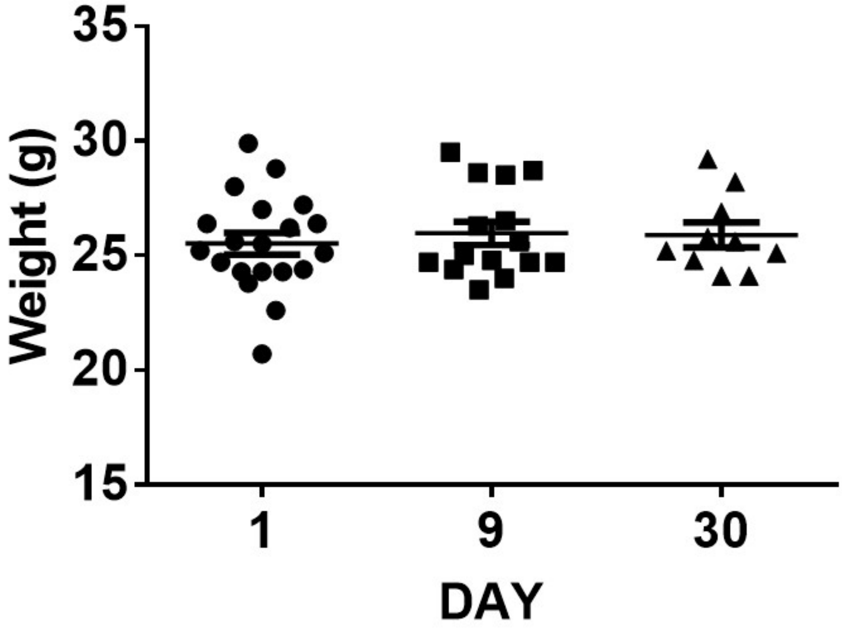

Figure S2. Cerium excretion in µg Ce/g tissue between day 1 and day 100.

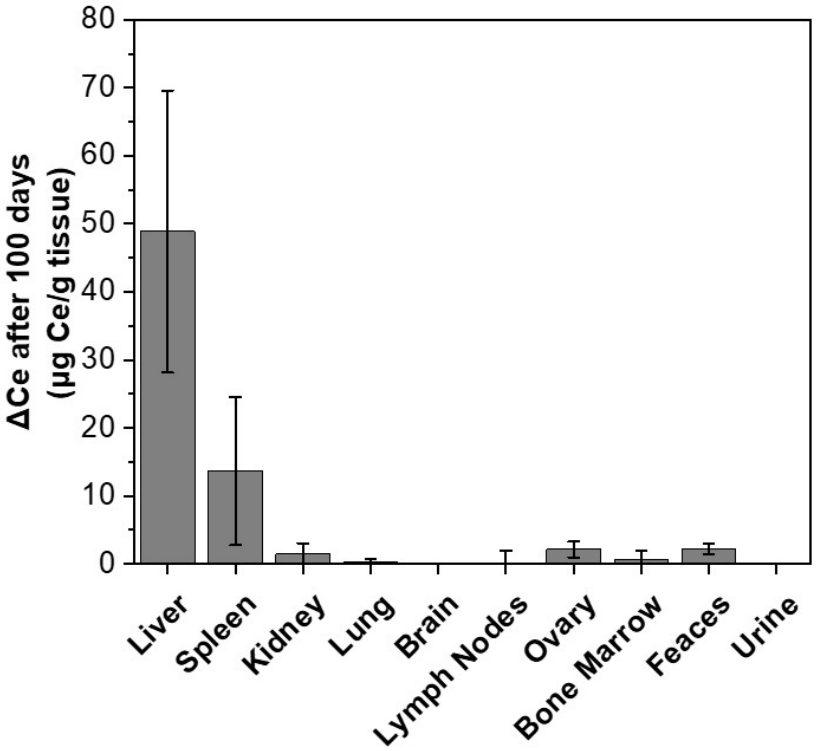

Figure S3. Dissolution of CeO_2 Nanoparticles at pH 1.7. Nanoceria was exposed to different pH 1.7. A decrease of UV-visible absorbance maximum with time confirms that NPs gradually dissolve. Complete dissolution can be observed after 3 hours.

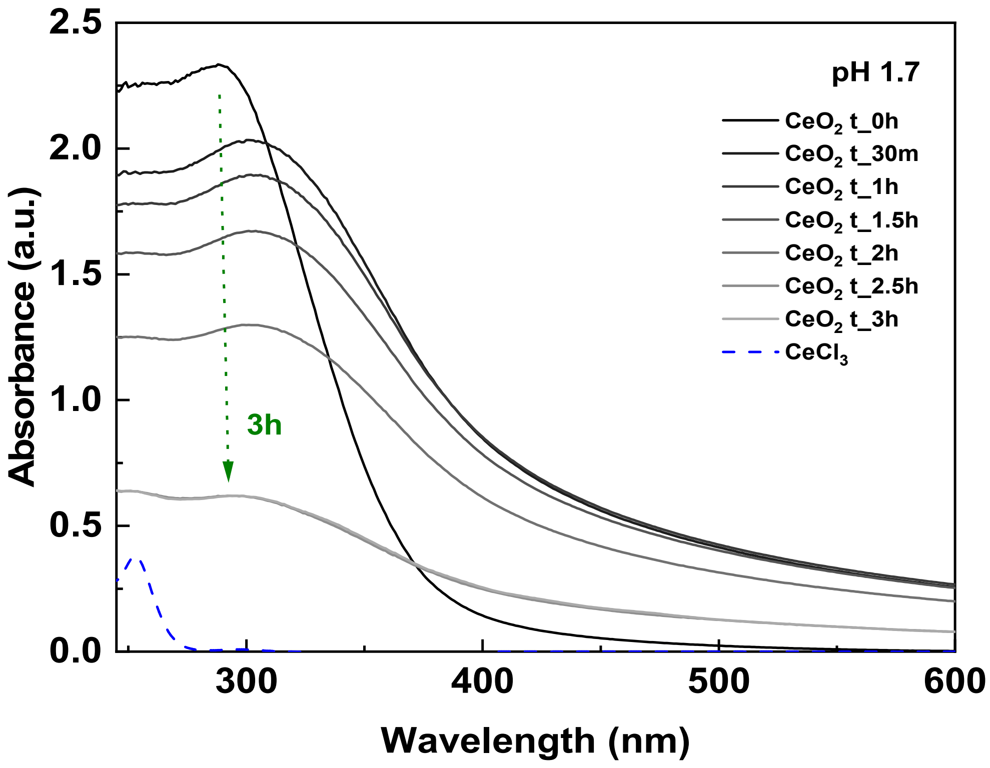

Figure S4. PourBoix diagram.

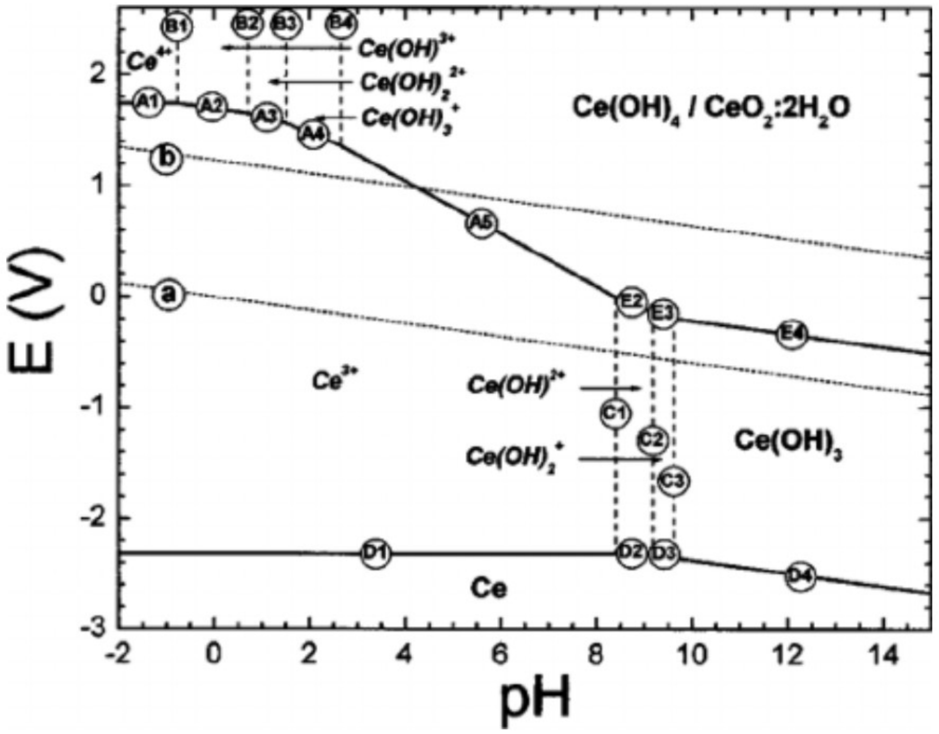

Figure S5. The liver and spleen are major targets for nanoparticles in CCl_4-treated rats. *In vivo* biodistribution of Fe_3O_4NPs in magnetic resonance imaging (MRI) axial abdomen and brain slices of a CCl_4-treated rat. The images on the left were taken from a representative CCl_4-treated rat. The images on the right were obtained from the same rat 90 minutes after NPs administration. Liver (L), spleen (S) and, at a much lower intensity, the kidney (K) are shown in black after NPs administration, indicating where Fe_3O_4NPs are accumulated. No changes were observed in brain.

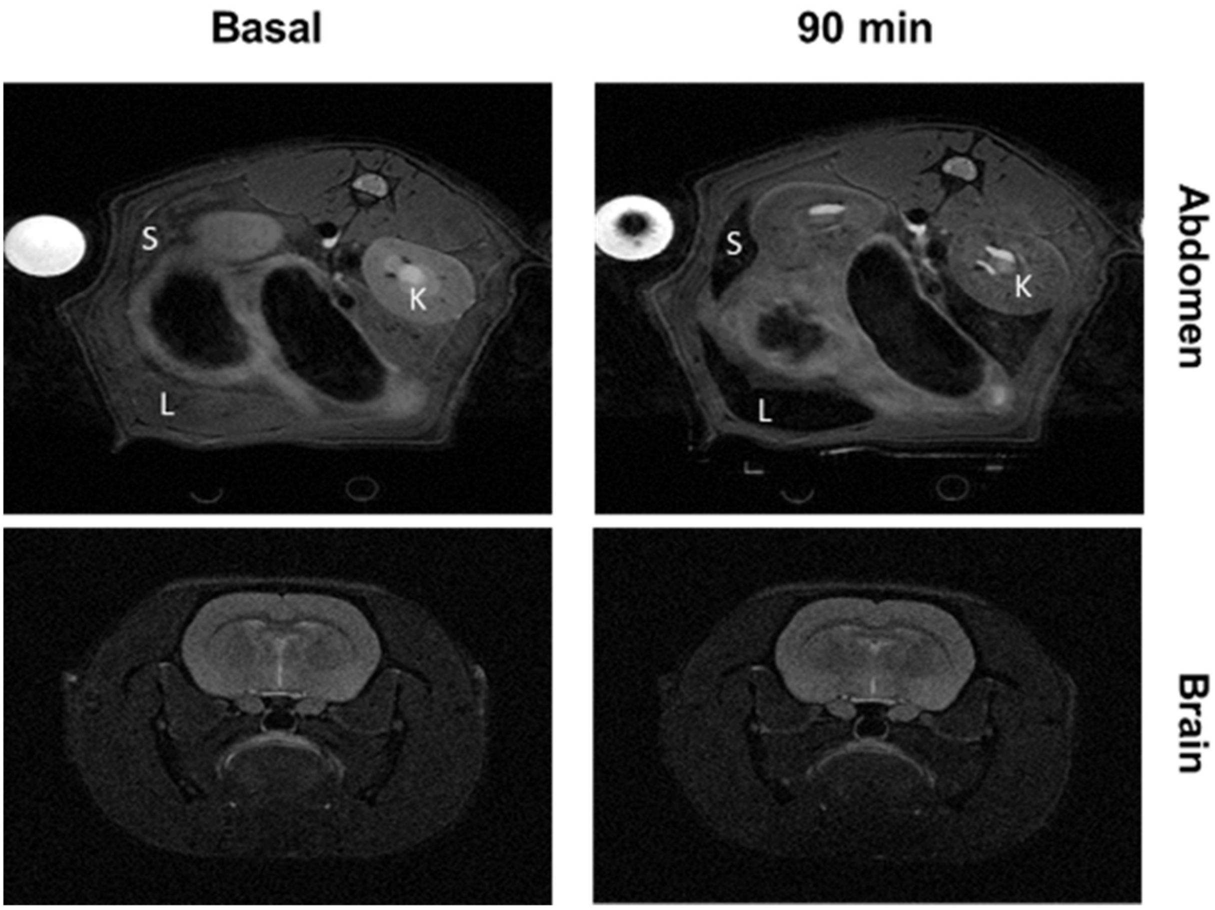

Extended Materials and Methods.

Fitting with traditional PK models. Experimental data were compared with the two principal pharmacokinetic models, the one-compartment and two-compartment models.

The one-compartment model assumes that the NPs are rapidly and uniformly distributed throughout the body and that the rate of NPs elimination is proportional to the amount of drug in the body. Therefore, the mathematical equation for a one-compartment model is:

$$C = Ae^{-k_1 t}$$

where C is the concentration of the NPs in the body, A is a constant that depends on the dose and volume of distribution, k_1 is the elimination rate constant, and t is the time after administration.

A two-compartment model assumes that the NPs have distributed into two compartments: a central compartment (such as blood) and a peripheral compartment (such as tissues). The mathematical equation for a two-compartment model is:

$$C = Ae^{-k_1 t} + Be^{-k_2 t}$$

where C is the NPs concentration in the body, A a constant that depends on the amount of NPs in the central compartment and the volume of the central compartment, k1 is the rate constant for distribution from the central compartment to the peripheral compartment, and t is time. B is a constant that depends on the number of NPs in the peripheral compartment and the volume of the peripheral compartment, and k2 is the rate constant for elimination from the peripheral compartment.

In both models, the concentration of the drug in the body can be predicted based on the dose and elimination rate constant. These models help predict NPs concentrations over time and optimize drug dosing regimens.

Obtained results from the fitting.

LIVER	ONE compartment model
	y = A1*exp(-x/t1) + y0
y0	43.65978 ± 3.34464
A1	57.96516 ± 18.53957
t1	4.35885 ± 2.39199
Reduced Chi-Sqr	0.60577
R-Square (COD)	0.93092
Adj. R-Square	0.79276

LIVER	TWO compartment model
	y = A1*exp(-x/t1) + A2*exp(-x/t2) + y0
y0	40.8684 ± 0
A1	26.12659 ± 0
t1	13.78216 ± 0
A2	26.12659 ± 0
t2	16.84486 ± 0
Reduced Chi-Sqr	0
R-Square (COD)	0.41196
Adj. R-Square	0

TOTAL	ONE compartment model
Equation	y = A1*exp(-x/t1) + y0
y0	2.74302 ± 0.21221
A1	2.77482 ± 0.97116
t1	5.69658 ± 5.16136
Reduced Chi-Sqr	1.83185

R-Square (COD)	0.92074
Adj. R-Square	0.76223

TOTAL	TWO compartment model
	y = A1*exp(-x/t1) + A2*exp(-x/t2) + y0
y0	2.56207 ± 0
A1	1.34301 ± 0
t1	14.70322 ± 0
A2	1.34301 ± 0
t2	17.9706 ± 0
Reduced Chi-Sqr	0
R-Square (COD)	0.86235
Adj. R-Square	0

Organ distribution of Fe_3O_4NPs in CCl_4-treated rats. To assess the tissue distribution of NPs, equivalent Fe_3O_4NPs were intravenously administered to 3 CCl_4-treated rats. Contrary to CeO_2NPs, Fe_3O_4NPs possess magnetic resonance activity. It has been shown how the NPs distribution is mainly dictated by size, shape, surface charge and surface state. In this case NPs were similar in morphology and both, after exposure to serum get coated with albumin resulting in equivalent surface and charge. Once 5 min of CCl_4 administration was reached the animals received Fe_3O_4NPs (2 mg/Kg bw) and CCl_4 treatment was maintained thereafter. Magnetic resonance imaging (MRI) was performed at 30 and 90 min and at 4 and 8 weeks following the Fe_3O_4NPs administration. The MRI experiments were conducted on a 7.0 T BioSpec 70/30 horizontal animal scanner (Bruker BioSpin, Ettlingen, Germany) equipped with a 12-cm inner diameter actively shielded gradient system (400 mT/m). The receiver coil was a phased-array surface coil for the rat brain. The animals were placed in a prone position in a Plexiglas holder with a nose cone to administer anesthetic gases (isoflurane in a mixture of 30% O_2 and 70% CO_2) and were held in position using tooth and ear bars, and adhesive tape. Tripilot scans were used for accurate positioning of the animal's liver in the isocenter of the magnet. T2-weighted images were acquired using a TurboRARE (rapid acquisition with rapid enhancement) sequence using the following parameters: repetition time= 3236 ms, echo time= 9 ms, RARE factor= 8, 4 averages, slice thickness= 1 mm with 1 mm gap between slices, number of slices= 60, field of view= 6 cm x 6 cm x 12 cm, matrix size= 256 x 256 x 60 pixels and a spatial resolution of 0.234 × 0.234 mm in a 1 mm slice thickness. To acquire the total volume of the abdomen, the acquisition was immediately repeated with the field of view shifted 1 mm in the z direction. The total scan time was 12 minutes. Images were processed and analyzed using the software ImageJ (National Institutes of Health, Bethesda, MD, USA).

Experimental procedure – Optical Microscopy of Tissue sections. 0.1 mL of 7 µg/mL of albuminized Au@SiO_2-FITC@PS NPs were intravenously injected via tail-vein injection (n=2). At 24 h, mice were euthanized via cervical dislocation and liver and organ samples were collected (including liver, spleen, and kidney). The organs were briefly washed with normal saline, slightly dried with blotting paper, and immersed for 4 h in 4% formalin for tissue fixation. Next, tissues were embedded in paraffin following a standard protocol. For immunohistochemistry, paraffin-

embedded samples were sectioned at 4 µm using a microtome and transferred to a poly-lysine coated glass slide. The paraffin was then removed with xylene and the samples rehydrated.

Immunostaining. Samples were permeabilized with Triton-X 0.2% for 10 min. Sections were incubated with anti-β-Actin mouse monoclonal antibody (MAB8929, R&D Systems, at a dilution 1:100) overnight at 4 °C, followed by an incubation with a secondary antibody goat anti-mouse Alexa Fluor568 (FisherScientific, A11019, dilution 1:500). Nuclei were stained with Hoesch 3342 (FisherScientific, H1399, dilution 1:10000). Fading was controlled using the Prolong antifade mounting medium (11559306).

Confocal Laser Scanning Microscopy (CSLM). Samples were observed on the Confocal Laser Scanning Microscope (Zeiss LSM 980 Airyscan). Hoescht 33342 ($_{em}$350/$_{ex}$461), Alexa Fluor-568 ($_{ex}$578/$_{em}$603) and FITC ($_{em}$490/$_{ex}$525) were observed using the standard fluorescence mode and pre-defined settings.

Dissolution of nanoceria at acidic pH. Nanoceria (500 μL, 1.72 mg/mL) was exposed to 2.85 mL of HCl. Final pH of the solution was 1.7. Aliquots were extracted at different time points as labelled and measured by UV-visible.
